# A Case of Biliary Peritonitis following Spontaneous Common Bile Duct Perforation in a Child

**DOI:** 10.5005/jp-journals-10018-1191

**Published:** 2016-12-01

**Authors:** Charu Sharma, Jayesh Desale, Mukta Waghmare, Hemanshi Shah

**Affiliations:** 1 Department of Paediatric Surgery, Topiwala National Medical College and B.Y.L. Nair CH. Hospital, Mumbai, Maharashtra, India

**Keywords:** Biliary peritonitis, Common bile duct, Laparotomy, Spontaneous perforation, Stenting.

## Abstract

Spontaneous common bile duct (CBD) perforation leading to biliary peritonitis is a rare entity in children. It is an unusual cause of acute abdomen and is potentially fatal. Preoperative diagnosis is rare. A 10-year-old girl was referred from a private hospital after laparotomy for acute abdomen. Intraoperatively, bile was found in the peritoneal cavity which was drained, and the patient was referred to Topiwala National Medical College. Endoscopic retrograde cholangiopancreaticography (ERCP) and stenting was done. The stent was removed after 8 weeks, and the girl is doing well during follow-up.

**How to cite this article:**

Sharma C, Desale J, Waghmare M, Shah H. A Case of Biliary Peritonitis following Spontaneous Common Bile Duct Perforation in a Child. Euroasian J Hepato-Gastroenterol 2016;6(2):167-169.

## INTRODUCTION

In the pediatric age group, there are multiple causes of common bile duct (CBD) perforation including spontaneous, or idiopathic, anomalies of pancreaticobiliary ductal system, congenital weakness of CBD, trauma, choledochal cyst, viral infection, stenosis of CBD, necrotizing enterocolitis, intramural thrombosis, and iatrogenic or stone in the CBD. The cause is idiopathic or spontaneous once the causes like choledochal cyst and trauma are ruled out.^1^ The peak age of occurrence is 6 months, with age ranging from 25 weeks of gestation to 7 years.^2^ The first case of this nature was described by Dijkstra in 1932 (compiled in ref. 2). Since then, about 150 cases have been reported, mostly in infants.^2^ The authors report a case of spontaneous CBD perforation causing biliary peritonitis in a 10-year-old girl who was managed by endoscopic retrograde cholangiopancreaticography (ERCP) and CBD stenting.

## CASE REPORT

**Fig. 1:**
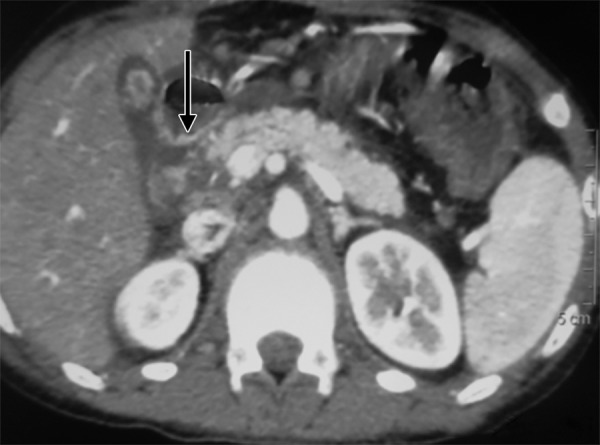
Contrast enhanced computed tomography scan showing an ill-defined collection in the Morrison’s pouch and mild free fluid in the peritoneal cavity

**Fig. 2:**
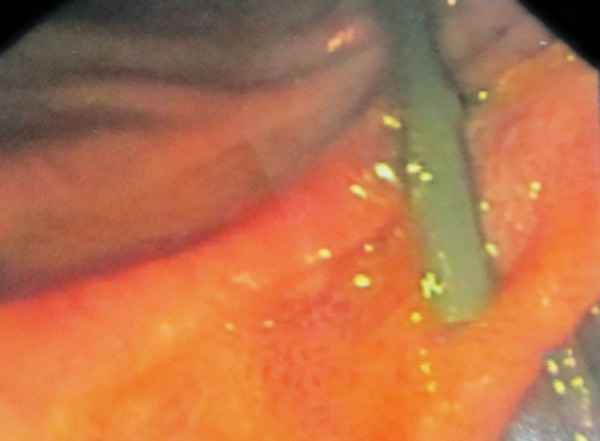
Endoscopic retrograde cholangiopancreaticography showing cannulation of the papilla

**Fig. 3:**
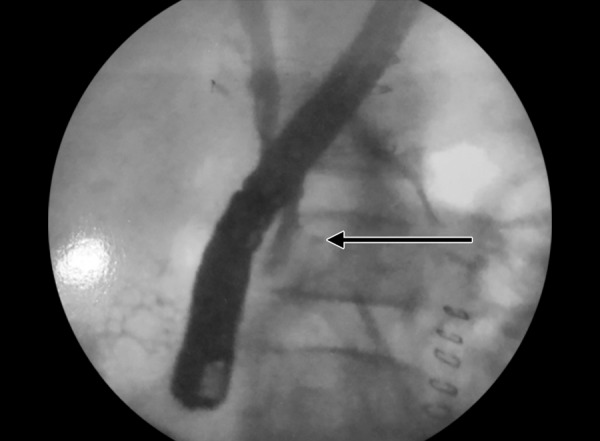
Cholangiogram showing CBD discontinuity and bile extravasation

A 10-year-old girl was admitted in a private hospital for an acute abdomen. She presented with complaints of abdominal pain, distention, and fever. Preoperative ultrasound was suggestive of free fluid. Intraoperatively, there was bile in the peritoneal cavity, no bowel perforation and a rent in CBD, suggestive of biliary tract perforation. A drain was kept in the Morrison’s pouch, and she was referred to our institute for further management. At admission, the patient was stable in regard to hemodynamical parameters. The draining output was approximately 200 to 300 mL of bile per day. Contrast enhanced computed tomography (CECT) scan suggested discontinuity in the medial wall of suprapancreatic CBD extending proximally for a length of approximately 1.4 cm with an ill-defined collection in the Morrison’s pouch and mild free fluid in the peritoneal cavity (Fig. 1). Endoscopic retrograde cholangiopancreatography revealed a leak from CBD just below the insertion of cystic duct (Figs 2 and 3), and a 7 French stent was inserted with the tip beyond the leak. The abdominal draining was stopped and removed. The patient was gradually shifted to oral diet. She was discharged after 8 days, and her stent was removed after 8 weeks. She is doing well on follow-up.

## DISCUSSION

Common bile duct perforation is a rare entity in the pediatric age group when compared with the adult population. In pediatric patients, the causes are varied and include spontaneous, or idiopathic, anomalies of pancreaticobiliary ductal system, congenital weakness of CBD, trauma, choledochal cyst, viral infection, stenosis of CBD, necrotizing enterocolitis, intramural thrombosis, and iatrogenic or stone in the CBD.^3^

The etiology of spontaneous perforation of CBD is unknown, but the proposed theories include congenital mural weakness of the CBD, ischemia, distal biliary obstruction, pancreaticobiliary malunion, and infection. Mostly, the diagnosis is made intraoperatively. Preoperatively, the diagnosis should be suspected when there is bilious abdominal paracentesis and when there are signs of peritonitis and absence of free gas in abdominal X-ray.^2^ Spontaneous perforation of the bile duct has also been associated with a multiple organ disorder, known as Ivemark syndrome, which consists of splenic abnormalities, cardiac pathology, and abnormalities of the gastrointestinal tract.^2,4^

The presentation of CBD perforation in children can be acute or insidious, with the latter presentation being more common (80%).^2^ Acute presentation includes fever, severe abdominal pain; vomiting; and signs of fulminant peritonitis, toxemia, and shock, resembling hollow viscous perforation. The insidious presentation includes progressive jaundice, painless abdominal distention, and clay-colored stools.^3^ The condition presents a diagnostic dilemma because of its rarity and absence of characteristic diagnostic finding; therefore, a high index of suspicion is required.^2^ Literature depicts only few cases in which the preoperative diagnosis was possible.^3^

Preoperative diagnosis, as previously stated, is difficult and various imaging modalities like abdominal ultrasound, computed tomography (CT) scan, magnetic resonance imaging (MRI), and radionuclide scan aid in the diagnosis.^3^ Laboratory evaluations are not pathognomonic. Conjugated bilirubin and alkaline phosphatase levels may be elevated. An ultrasound will show free or loculated intraperitoneal fluid with normal intra- and extrahepatic ducts.^2^ Paracentesis carried out under ultrasonography guidance may reveal bile-stained fluid.^2^ In the majority of cases, biliary tract injuries are diagnosed intraoperatively. Endoscopic retrograde cholangiopancreatography, when available, is an important diagnostic and therapeutic tool. Hepatobiliary scintigraphy can show that the intraperitoneal fluid originated from the biliary tract. It is highly sensitive and specific for spontaneous perforation of CBD.^2,5^

Drip infusion cholangiography using meglumine iotroxate is useful in pancreaticobiliary maljunction, billiary stricture, and perforation. Finally, peritonitis with absence of pneumoperitoneum, bilious peritoneal tap, and acholic stool are considered pathognomonic for spontaneous bile duct perforation.^2^

Various approaches for the management of pediatric CBD perforations have been described in literature, but these need to be tailored according to the general condition of the patient, the extent of the peritonitis, and the imaging findings. Patients presenting with generalized peritonitis require surgical exploration, thorough lavage and drainage of the peritoneal cavity, management of the perforation per se, and treatment of any associated biliary pathology.^6,7^ The perforated CBD can be managed by simple drainage at the site of perforation, with or without biliary diversion, and with or without closing the perforation. A perforation may also be closed over a T-tube if there is no associated biliary pathology.^3,7^ Suture repair may not be possible if severe inflammation is present at the perforation site. A more proximal perforation of the hepatic duct may also preclude the feasibility of primary repair. These patients may be managed with biliary decompression with T-tube drainage provided there is no distal obstruction. Previously, cholecystectomy with CBD excision and Roux-en-Y hepaticoenterostomy was done for CBD perforation. But now, it is preferred for nontreatable distal obstruction, persistent biliocutaneous fistula, persistent biliary leak, and choledochal cyst-associated CBD perforation.^3^

Exploration of the porta hepatis may be hazardous at emergency, and therefore, simple peritoneal drainage with T-tube drainage is recommend even if there is a distal obstruction. This entails less morbidity and has a good chance of cure of the condition or at least stabilizing the patient for second-look definitive surgery.^2,5^ Pancreaticobiliary malunion requires biliary intestinal anastomosis to prevent biliary cirrhosis, portal hypertension, recurrent pancreatitis, and ultimately biliary carcinoma.^2,5^ However, this can be done at a second laparotomy when inflammation has settled. Repair of the perforation is unnecessary and may be hazardous because there is also the risk of postoperative stricture. With recent advances in laparoscopic surgery, diagnosis and percutaneous drainage is the alternative.^2^

The timing of biliary stent removal has been variable in previous reports of bile duct perforation. Barnes et al^8^ reported a case of spontaneous bile duct perforation in a 3-year-old toddler. They managed the case with endoscopic biliary stenting and removed the stent successfully after 7 weeks of the procedure.^6^

The postoperative complications of biliary peritonitis are wound infection, burst abdomen, persistent peritonitis, prolonged ileus, hemobilia, gallbladder necrosis, etc.^3^ These can be reduced by ERCP, as in our case. The previous high mortality rates have been reduced to almost zero in most recent studies,^3^ the main reasons being early intervention and development of better intensive care modalities.

## CONCLUSION

Biliary peritonitis due to spontaneous CBD perforation in pediatric age group is an uncommon but manageable condition. Presentation may be acute or insidious, preoperative diagnosis is difficult. Endoscopic retrograde cholangiopancreatography acts as a diagnostic and therapeutic tool and avoids operative exploration. Thus complications and mortality gets reduced.

## CLINICAL SIGNIFICANCE

This rare condition is difficult to diagnose and presents usually as acute abdomen. Endoscopic retrograde cholangiopancreatography is both diagnostic and therapeutic in the form of stenting and avoids laparotomy when available.
